# Surrogate gating strategies for the Elekta Unity MR‐Linac gating system

**DOI:** 10.1002/acm2.14566

**Published:** 2024-11-14

**Authors:** Samuel D. Rusu, Blake R. Smith, Joel J. St‐Aubin, Nathan Shaffer, Daniel Ellis Hyer

**Affiliations:** ^1^ Department of Radiation Oncology University of Iowa Hospitals and Clinics Iowa City Iowa USA

**Keywords:** Elekta Unity, gating, motion management, MR linac

## Abstract

**Purpose:**

MRI‐guided adaptive radiotherapy can directly monitor the anatomical positioning of the intended target during treatment with no additional imaging dose. Elekta has recently released its comprehensive motion management (CMM) solution that enables automatic radiation beam‐gating on the Unity MR‐Linac. Easily visualized targets that are distinct from the surrounding anatomy can be used to drive automatic gating decisions from the MRI cine imaging. However, poorly visualized targets can compromise the tracking and gating capabilities and may require surrogate tracking structures. This work presents strategies to generate robust tracking surrogates for a variety of treatment sites, enabling a wider application of CMM.

**Methods:**

Surrogate tracking strategies were developed from a cohort of patients treated using the CMM system on the Unity MR‐Linac for treatment sites of the lung, pancreas, liver, and prostate. These sites posed challenging visualization or tracking of the primary target thereby compromising the tracking accuracy. Surrogate structures were developed using site‐specific strategies to improve the imaging textured detail within the tracking volume while avoiding the dynamic overwhelming hypo‐ or hyper‐intense anatomical structures. These surrogate volumes were applied within the anatomical positioning monitoring system as a proxy that drove the CMM gating decisions on the treatment unit.

**Results:**

Robust site‐specific surrogate structures were developed. Surrogate tracking structures for centrally located thoracic targets were created by expanding the target peripherally away from the heart and great vessels and into the lung. Pancreas surrogates required a vertically expanded column intersecting with the inferior liver edge. For the liver and prostate, surrogate structures consisted of a uniform expansion of the target, with liver surrogates intersecting the proximal liver edge or diaphragm while avoiding nearby ribs.

**Conclusion:**

These surrogate strategies have enabled the gating of complex moving targets among different treatment sites at our institution.

## 1 INTRODUCTION

**FIGURE 1:**
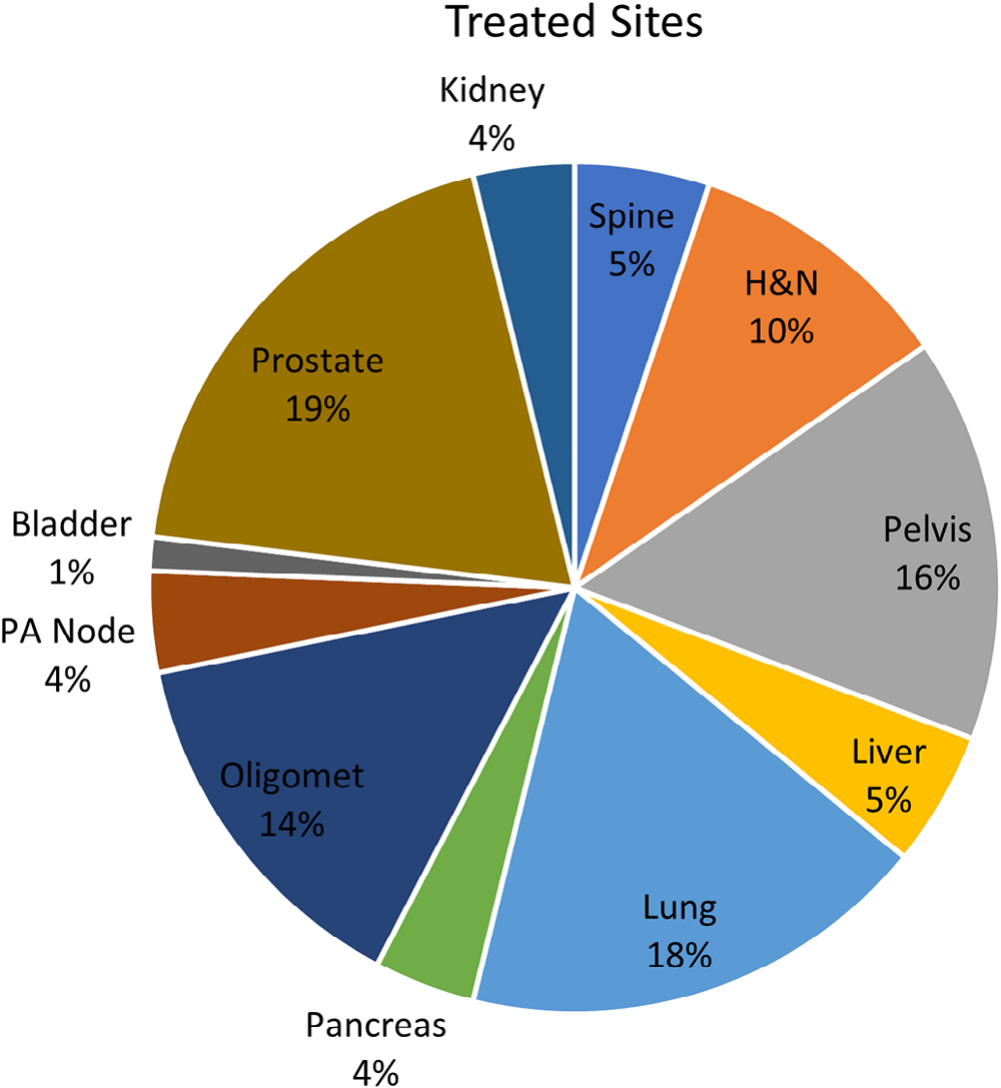
Our institution's treated sites using CMM and APM.

The Unity (Elekta AB, Stockholm, Sweden) is a hybrid 1.5 T MRI linac (MR‐linac) that has been used clinically since 2018 to provide adaptive radiotherapy to various treatment sites.^1^ MRI‐guided adaptive radiotherapy enables direct anatomical monitoring of the treatment using real‐time cine imaging with no additional imaging dose. As of February 2023, Elekta received FDA 510(k) clearance for the comprehensive motion management (CMM) package for the Elekta Unity, which enabled automatic beam gating capabilities using 3D position tracking based on live cine imaging with virtually zero system latency.^2^ CMM employs a validated template‐based registration algorithm for real‐time motion monitoring on the Unity^3^, ^4^ based on a user‐defined tracking structure, which may be defined as a surrogate of the intended target that would have otherwise been difficult to visualize. CMM also allows for drift correction in situations that the target moves above some limit, through baseline shifting, which can be useful in cases that motion is not expected but can occur. As one of the pilot sites, we have incorporated CMM for all treatments, treating both SBRT and conventionally fractionated gating cases using the novel anatomical position monitoring (APM) system with predictive tracking capabilities.^2^ A pie chart illustrating the treatment sites we have treated with CMM is presented in Figure 1.

Successful tracking surrogates have been reported for similar MR‐linac technologies, such as the MRIdian linac (Viewray, Oakwood Village, Ohio, USA). With the MRIdian linac, the gating target is automatically detected using deformable registration and is used for gating in combination with cross‐correlation thresholds between the live frame and the key frame to monitor bulk motion or anatomic changes.^5^ A more in‐depth comparison of CMM and other MR‐guided radiation therapy solutions has been presented in literature.^5^ Some of the internal surrogates used for lung and pancreas with this system are the abdominal surface or the diaphragm, or a combination of the two,^6^, ^7^, ^8^ the surface of the liver or prominent adjacent liver vessels,^9^, ^10^, ^11^, ^12^ and the rectum and bladder interface to track the prostate.^13^

While surrogate strategies exist for other MR‐Linac technologies reported from other institutions,^6^, ^7^, ^8^, ^9^, ^10^, ^11^, ^12^, ^13^ there are no published strategies or experiences for the Elekta Unity, which utilizes very different tracking algorithms, MR imaging sequences, and equipment. The aim of this work therefore is to share clinical strategies that were developed using custom surrogate structures at our institution to enable a wider application of this technology to more treatment sites and to save clinics time when implementing CMM or other MR‐based gating into their own clinics.

## 2 METHODS

The CMM package on the Unity MR‐Linac was used to treat patients using both SBRT and conventionally fractionated gating cases. A subset of four patients with tumors located in the central lung, pancreas, liver, and prostate requiring surrogate tracking structures are reported in this study. The use of patient data used for this project was reviewed by the IRB of Record (UIOWA IRB‐01, biomedical) and deemed not to meet criteria for human subjects research. Specific information regarding the treatment techniques used for each patient are listed in Table 1. Anatomical tracking for each patient was performed using a 2D balanced turbo field echo (bTFE) sequence, which yielded MR cine images acquired every 200 ms in alternating sagittal and coronal planes.^14^ Beam gating in CMM was performed using a Gating Envelope technique with a percent volume overlap threshold of 95%^3^, ^14^ based on the position of the target (GTV or CTV) and its overlap with the gating envelope (PTV). The motion/position of the target was inferred by the tracking surrogate.

**TABLE 1:** Site‐specific surrogate structure summary.

Site	Compression used	Fractionation	PTV margins used (cm)	Surrogate structure definition
Centrally located lung target SBRT	No	750 cGy × 8	0.2–0.3 cm on ITV	0.5–1.5 cm expansion of GTV **AVOID** the heart, aorta, and diaphragm (using a margin between 2 and 5 mm if needed)
Pancreas SBRT	Yes	700 cGy × 5	0.3–0.5 cm on ITV	Superior expansion of the GTV (pancreatic head) [ex. 3 cm] **INTERSECTED** with the liver edge
Liver SBRT	Yes	1800 cGy × 3	0.3–0.5 cm on ITV	Expansion [ex. 3 cm] of the GTV towards the proximal liver edge **AVOID** the region 5 mm expanded from the bone **INTERSECTED** with the liver edge
Prostate	No	275 cGy × 20	0.3–0.5 cm margin on prostate (except post) 0.2–0.4 cm posterior expansion	1 cm uniform margin on the CTV

**FIGURE 2:**
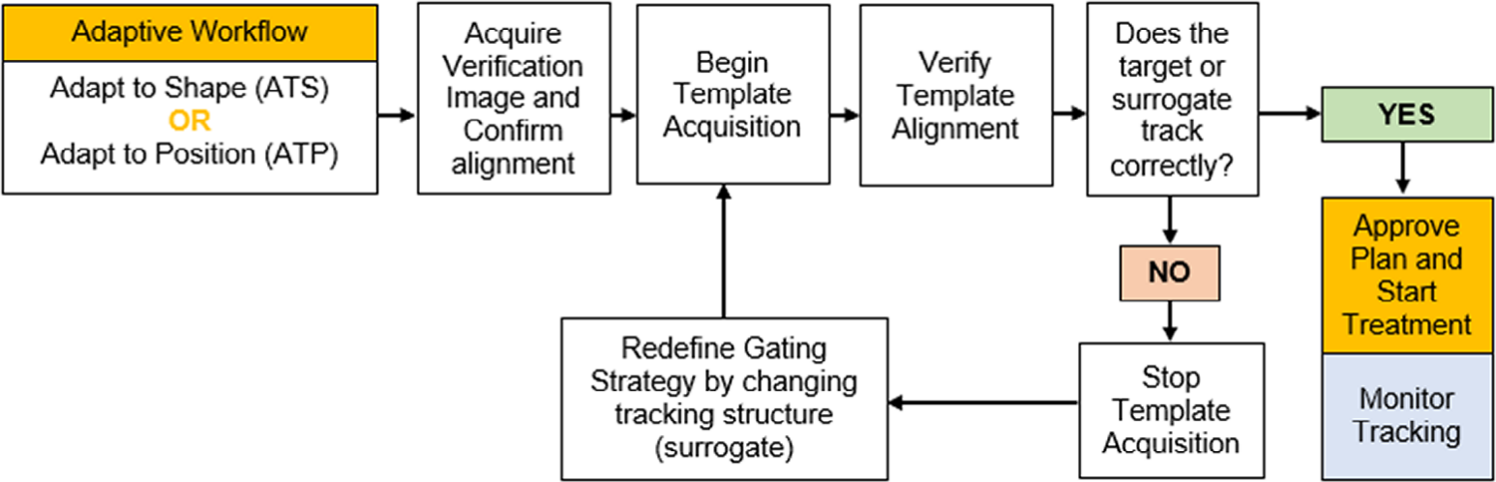
Our institution's workflow for gated treatments using CMM and APM.

The Elekta automatic gating tracking algorithm is comprised of three major components, specifically, quality factor metrics, tracking accuracy, and the automatic gating component to ensure gating errors are minimized. A successful beam‐on occurs only if all components are satisfied. If any of the quality or tracking accuracy metrics fail, then a beam inhibit prevents treatment. The quality factor metrics are used to detect problematic situations in 2D‐cine images such as large anatomy deformations, through‐plane motion, jitter, no‐motion, and drop in registration score detection.^5^ The tracking accuracy metric is a measure of the gating accuracy confidence based on the gating decisions made over the past 3.3 s of beam‐on time. Our current clinical process for determining the need for a surrogate strategy follows our site‐specific clinical experience as seen in the workflow presented in Figure 2 and is an expansion of clinical workflows presented in literature for the Unity.^15^ Surrogate structures balancing the size, MR signal, and the regional anatomy surrounding the target were developed using site‐specific strategies and applied within the anatomical positioning monitoring system as a proxy that drove gating decisions.

## 3 RESULTS

Site‐specific surrogate structures were developed and a summary of how they were generated is presented in Table 1. All surrogate structures were generated as auto‐margins on already existing contours in the plan. To determine the specific margins chosen to generate the surrogate structures, one must balance the target size, target motion, the MR signal, and the regional anatomy surrounding the target. As a general rule of thumb, margins used should avoid hypo/hyper intense anatomy that change frame to frame during cine imaging or bones that are not expected to move with the target. The surrogate structure should also move rigidly with the tracked target since the surrogate structure will be used for determining the target motion. These recommendations can often be satisfied with an equally expanded margin around the target or an expanded margin toward an anatomical edge or boundary that is easily detectable and tracked on the cine. Another challenge one must consider when defining margins is that the center of the surrogate structure is used for both template acquisition validation and to define the planes of the cine MR imaging. As a result, if the surrogate comprises concave features or is an asymmetric expansion of the target, the planes used for tracking might not include the target to be tracked, limiting the usefulness of the system. If the target is not visible, this is not of concern. Therefore, a good surrogate structure moves rigidly with the target and allows for correct tracking of the target resulting in accurate and predictable gating decisions.

All surrogate structures developed were used clinically as shown in the workflow presented in Figure 2. The magnitude of the distances suggested are sample expansions and should be customized as required for the user's need. For the sites presented here, the surrogate resulted in stable intra‐ and inter‐fraction tracking using balanced turbo field echo cine sequences. We suggest that for similar situations as presented, a surrogate structure be used. The tracking strategies developed from these clinical experiences are presented in the following section.

### 3.1 Centrally located lung targets

**FIGURE 3:**
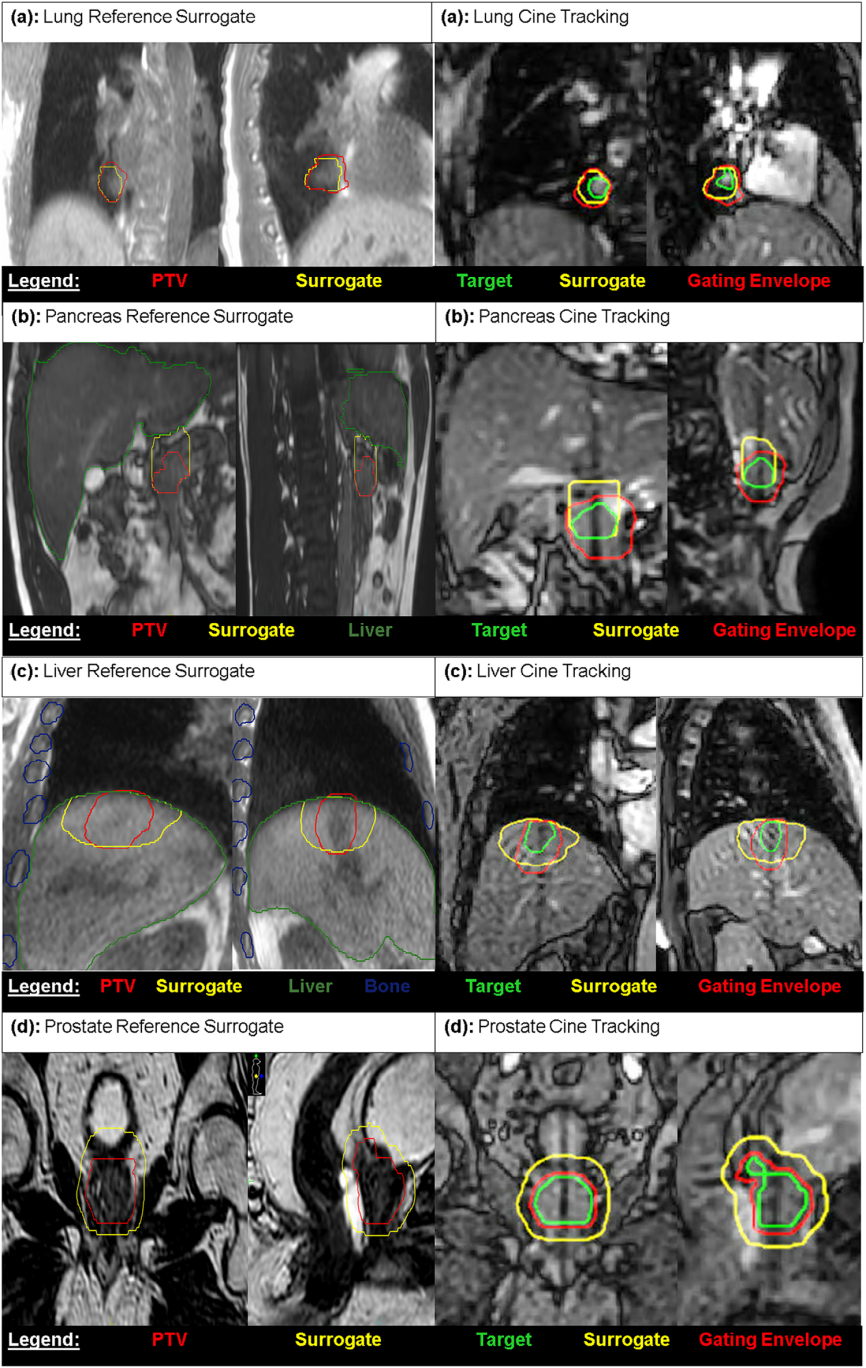
Reference and Cine Imaging tracking planes for the lung (a), pancreas (b), liver (c), and prostate (d). The GTV is the defined as the target, the PTV is the gating envelope, a 95% volume overlap threshold is used, and the surrogates are displayed for reference.

For centrally located lung targets, the heart and aorta exhibited a notable hyperintensity in the bTFE cine sequence due to the rapidly changing blood flow. As such, directly tracking the target is difficult and resulted in inconsistent results which would inhibit the treatment. As a solution, a surrogate was created using a 0.5–1.5 cm expansion of GTV into the ipsilateral lung avoiding the heart, aorta, and diaphragm but including smaller vessels in the lung (Figure 3a). Depending on the situation, using a margin on the avoided structures of 2–5 mm was also found to be advantageous. This surrogate was found to track the motion of the tumor reliably. Using expansions in the range given above to define the surrogate has proven to be robust for multiple patients providing stable intra‐fraction and inter‐fraction tracking.

### 3.2 Pancreas

Treating the pancreatic head is challenging since it is a target that has poor contrast on bTFE imaging and thus is poorly differentiated from the surrounding tissue. To track the target, a tracking surrogate structure was generated using a 3 cm superior expansion of the GTV (pancreatic head) intersected with the liver edge (Figure 3b). The liver edge provided sufficient contrast for the tracking algorithm to track the head of the pancreas.

### 3.3 Liver

Small liver targets with no contrast can be very challenging to visualize on MRI making them extremely difficult to track. Regardless of the location of the target, having some expansion that intersects the proximal liver edge worked best based on our experience. It is recommended that for the surrogate, one can expand the GTV to the proximal liver edge avoiding bone. As an example, a surrogate was created by adding a 3 cm expansion from the GTV (toward the nearest liver edge), avoiding bone (plus a 5 mm expansion), and intersected with the liver edge (Figure 3c). Bone structures are specifically avoided in the region of tracking, as it is a rigid stationary structure that does not move with respiratory targets and can result in poor tracking.

### 3.4 Prostate

Bladder and rectum intra‐fractional changes during the course of treatment complicated direct tracking of the prostate due to the T2/T1‐weighted contrast on the balanced contrast MR cine imaging. Thus, a surrogate was created using a 1 cm uniform margin on the CTV, which allowed for more consistent tracking compared to tracking the prostate alone.

### 3.5 Surrogate results

The surrogate strategies reduced erroneous inhibits due to poor quality factor metrics or low accuracy tracking. A tracking success rate based on the quality factor for the surrogate structures was found to be 98.9%, 95.1%, 98.0%, 98.9% for the representative lung, prostate, pancreas, and liver cases, respectively.

## 4 DISCUSSION

The information presented in this work is based on our initial clinical experiences, and as such, an investigation of multiple surrogate strategies was not possible due to the in‐vivo and time‐sensitive nature of this work. Direct comparison to the conventional use of the target without a surrogate structure is also difficult, as tracking the nominal target was not a viable option. For example, tracking the head of the pancreas or non‐visualizable liver tumor resulted in continuous quality and tracking error inhibits from CMM.

If the target is not visible, if it is near hypo/hyper intense changing anatomy, or the balanced contrast on the MR cine imaging is not ideal, we recommend that some auto‐margin generated surrogate structure be used to provide robust tracking for intra‐ and inter‐fraction daily treatment deliveries. If the target is visible and trackable, a surrogate might not be necessarily needed. However, for targets that are not visible, we recommend developing a surrogate and following the workflow presented in Figure 2.

The strategies presented in this work performed consistently intra‐ and inter‐fractionally using CMM system. Practically, using the workflow presented in Figure 2, different surrogate structure strategies can still be tested in an online setting. After a surrogate structure is generated, the APM system can be initialized and evaluated before approving the treatment plan. If low‐accuracy or low‐quality inhibits are present, or the surrogate strategy using regional anatomy around the target appears to be poorly tracked, a different surrogate strategy can then be attempted. The margins presented are representative examples of margins used for surrogate generation and are not exclusive. Other margins can be used, and personalized target margins depending on the site are encouraged.

## 5 CONCLUSION

Surrogate gating strategies for tracking targets that posed particularly challenging visualization at our institution for treatment sites in the lung, pancreas, liver, and prostate were presented. Using these surrogate structures, strategies and the workflow presented allowed for the gating of complex moving targets among different treatment sites.

## AUTHOR CONTRIBUTIONS

All authors were part of the design of the surrogate gating strategies, tested the surrogate structures and gating strategies clinically, helped in the development of the manuscript, editing, drafting of the study, and approved the final version to be published.

## CONFLICT OF INTEREST STATEMENT

Joel St‐Aubin reports honorarium and research funding from Elekta unrelated to this work. Daniel Hyer discloses a consulting relationship with Elekta and research funding from Elekta unrelated to this work. The remaining authors have no conflicts of interest to disclose.
